# The Transcriptome of *Legionella pneumophila*-Infected Human Monocyte-Derived Macrophages

**DOI:** 10.1371/journal.pone.0114914

**Published:** 2014-12-08

**Authors:** Christopher T. D. Price, Yousef Abu Kwaik

**Affiliations:** 1 Department of Microbiology and Immunology, University of Louisville, KY, 40202, United States of America; 2 Center for Predictive Medicine, University of Louisville, KY, 40202, United States of America; University of Arkansas for Medical Sciences, United States of America

## Abstract

**Background:**

*Legionella pneumophila* is an intracellular bacterial pathogen that invades and replicates within alveolar macrophages through injection of ∼300 effector proteins by its Dot/Icm type IV translocation apparatus. The *bona fide* F-box protein, AnkB, is a nutritional virulence effector that triggers macrophages to generate a surplus of amino acids, which is essential for intravacuolar proliferation. Therefore, the *ankB* mutant represents a novel genetic tool to determine the transcriptional response of human monocyte-derived macrophages (hMDMs) to actively replicating *L. pneumophila*.

**Methodology/Principal Findings:**

Here, we utilized total human gene microarrays to determine the global transcriptional response of hMDMs to infection by wild type or the *ankB* mutant of *L. pneumophila*. The transcriptomes of hMDMs infected with either actively proliferating wild type or non-replicative *ankB* mutant bacteria were remarkably similar. The transcriptome of infected hMDMs was predominated by up-regulation of inflammatory pathways (IL-10 anti-inflammatory, interferon signaling and amphoterin signaling), anti-apoptosis, and down-regulation of protein synthesis pathways. In addition, *L. pneumophila* modulated diverse metabolic pathways, particularly those associated with bio-active lipid metabolism, and SLC amino acid transporters expression.

**Conclusion/Significance:**

Taken together, the hMDM transcriptional response to *L. pneumophila* is independent of intra-vacuolar replication of the bacteria and primarily involves modulation of the immune response and metabolic as well as nutritional pathways.

## Introduction

*Legionella pneumophila* is found ubiquitously in the aquatic environment and shares an intimate intracellular relationship with many species of amoeba and ciliates [1], [2], [3]. *L. pneumophila*, the causative agent of Legionnaires' disease, invades and replicates in human alveolar macrophages [4], [5]. When *L. pneumophila* invades amoeba or human macrophages, it evades the default endosomal-lysosomal degradation pathway and remodels its phagosome into a specialized ER-derived vacuole via intercepting ER-to-golgi vesicular trafficking [2], [3], [5], [6]. This is achieved by the translocation of ∼300 effector proteins via the Dot/Icm type IVB secretion system [5], [7], [8], [9]. These effectors modulate a myriad of eukaryotic processes including host signaling, vesicular trafficking, protein synthesis, apoptosis, prenylation, ubiquitination, and proteasomal degradation [2], [6], [10], [11], [12], [13]. Surprisingly, very few of these effectors are essential for intracellular replication of *L. pneumophila* [5], [14], suggesting specific requirements for different effectors in different environmental hosts.

The AnkB translocated effector is one of very few effectors essential for proliferation of *L. pneumophila* strain AA100/130B within the two evolutionarily-distant hosts, mammalian and protozoan cells, and for intrapulmonary bacterial proliferation and manifestation of pulmonary disease in the mouse model [15], [16], [17], [18], [19]. In addition, AnkB in *L. pneumophila* strain Paris contributes to intravacuolar proliferation in the THP-1 human macrophage cell line and in A549 human lung epithelial cells and is needed for lung colonization of A/J mice, albeit at a less extent than in strain AA100 [20]. In contrast, AnkB is dispensable for intravacuolar replication of *L. pneumophila* strain Philadelphia-derived Lp02 in macrophages [21], suggesting that a compensatory genetic repertoire exists in this strain that overcomes the loss of AnkB. AnkB is a non-canonical F-box protein that interacts with the host SCF1 ubiquitin ligase on the LCV membrane [22] and functions as a platform for the docking of Lys^48^-linked polyubiquitinated proteins to the *Legionella*-containing vacuolar (LCV) membrane [18], [20]. The AnkB-assembled Lys^48^-linked polyubiquitinated proteins are degraded by the host proteasome machinery, which generates higher levels of cellular amino acids [19]. These amino acids are used by *L. pneumophila* to feed the tri-carboxylic acid (TCA) cycle to generate ATP and secondary metabolites to power intra-vacuolar replication of *L. pneumophila* [19], [23], [24]. Since the only defect of the *ankB* mutant is its inability to import sufficient levels of host amino acids and is localized within an ER-derived LCV that evades lysosomal fusion similar to the wild type strain [15], the *ankB* mutant is a useful genetic tool to probe the global human macrophage responses to actively replicating *L. pneumophila*.

The global transcriptional profile of *L. pneumophila*-resistant bone marrow-derived C57BL/6J and the congenic *L. pneumophila*-susceptible BcA75 murine macrophages in response to *L. pneumophila* infection revealed striking host modulation of gene expression [25], [26]. C57BL/6J mouse macrophages are inherently resistant to *L. pneumophila* because the inflammasome is activated through Naip5-dependent sensing of bacterial flagellin [27], [28], [29], [30], [31]. In contrast, A/J mice have an altered Naip5 allele that renders this mouse strain sensitive to *L. pneumophila* infection. The C57BL/6J congenic mouse strain BcA75 harbors the A/J Naip5 allele [25]. The transcriptional profile of bone marrow-derived macrophages (bMDMs) isolated from C57BL/6J or BcA75 mice in response to *L. pneumophila* infection are very similar, indicating that the mouse macrophage transcriptional response is independent of inflammasome activation [25]. Further transcriptome studies using C57BL/6J bMDMs, revealed induction of a novel innate immune response termed the ‘effector triggered response’ (ETR) [26] that is dependent on five *L. pneumophila* effectors that directly block host protein translation [32], [33], [34], [35], [36]. In addition to this finding, recent work has demonstrated that infected cells exhibit a frustrated MAP kinase response due to effector-dependent host protein synthesis inhibition, but inhibition of pro-inflammatory cytokine translation is overcome in these cells by a MyD88-dependent mechanism [37]. Additional work demonstrated the global transcriptional response of the human macrophage-like U937 cell line to a low dose challenge of *L. pneumophila* and found up-regulation of anti-apoptotic genes controlled by the transcriptional regulator NF-κB [38]. However, genome wide global transcriptome analysis of primary human monocyte-derived macrophages (hMDMs), the mammalian host cell for *L. pneumophila*, has not been evaluated. Analyzing the global transcriptome response of hMDMs to *L. pneumophila* infection is important to determine the cellular responses in the human host and would allow us to compare the findings to the model system of bMDMs of the mouse animal model.

Here we show the global transcriptional response of hMDMs infected with wild type *L. pneumophila* and its isogenic *ankB* mutant. The *ankB* mutant was selected for this study because it fails to replicate intracellularly, but is enclosed within an ER-derived vacuole that evades lysosomal fusion and translocate its full repertoire of effectors, similar to the wild type strain. It is a unique nutritional mutant that resides within a replicative vacuole but simply lacks sufficient levels of amino acids to power its proliferation. Interestingly, the hMDM transcriptional response to *L. pneumophila*, involves modulation of several immunological pathways, protein synthesis pathways, metabolic pathways and amino acid transporters. Importantly, the global response of hMDMs to infection by *L. pneumophila* is independent of intracellular bacterial replication.

## Materials and Methods

### Ethics statement

Human monocyte-derived macrophages were obtained from healthy donors who had given written consent for their use, and this work was approved by the University of Louisville Institutional Review Board.

### Bacterial strains and cell cultures

*L. pneumophila* strain AA100/130b (ATCC BAA-74) and the isogenic *ankB* mutant were grown on BCYE agar plates for 3 days at 37°C prior to use for infection of macrophages [15]. Human monocyte-derived macrophages (hMDMs) were isolated from healthy donors as described previously IRB [18]. Briefly, monocytes were isolated from whole blood and allowed to adhere to low adherence cell culture plates for 3 days in RPMI 1640 supplemented with 20% FBS at 37°C and 5% CO_2_. The monocytes were then counted and resuspended RPMI 1640 supplemented with 10% FBS and plated at a density of 3×10^6^ cells per well of a 6 well cell culture plate and incubated for a further 2 days. The cell culture media was then replaced with RPMI 1640 supplemented with 5% FBS for one day, and then with RPMI 1640 supplemented with 1% FBS for one day. The mature hMDMs were then used for infection by *L. pneumophila*.

### Isolation of RNA from *L. pneumophila* infected hMDMs

The hMDM monolayers plated at density of 3×10^6^ cells per well in a 6 well plate were either uninfected or infected in triplicate with either the wild type *L. pneumophila* or the *ankB* mutant at an MOI of 20 for 1 h and then treated for 1 h with gentamicin to kill remaining extracellular bacteria and incubated at 37°C and 5% CO_2_. The infections were allowed to proceed for a total of 8 h prior to isolation of total RNA. Total RNA from the hMDM monolayers was isolated from 3 individual wells for each condition (uninfected, wild type or *ankB* infected) using the Qiagen RNeasy Mini kit (Qiagen) according to the manufacturer's instructions. A total of 3 biological replicates were performed. Quality of the purified RNA from each sample was confirmed by using a Bioanalyzer (Agilent) prior to use for microarray analysis.

### Microarray sample preparation and analysis

Total RNA was amplified from each sample and labeled following the Affymetrix (Santa Clara, CA) standard protocol for whole transcript expression analysis followed by hybridization to individual Affymetrix Human Gene 1.0 ST arrays for each sample. The arrays were processed following the manufacturer recommended wash and stain protocol on an Affymetrix FS-450 fluidics station and scanned on an Affymetrix GeneChip 7G scanner using Command Console 3.1. The resulting.cel files were imported into Partek Genomics Suite 6.6 and transcripts were normalized on a gene level using RMA as normalization and background correction method. Contrasts in a 1-way ANOVA were set up to compare the treatments of interest. The data was then analyzed using Metacore Pathway software (Thomson Reuters) with a threshold set to set to 0 and a *p*-value of 0.15. Microarray data was submitted to the Gene Expression Omnibus (GEO) repository and can be accessed through accession number GSE61535. This data set also contains additional microarray data of hMDMs infected with a type II secretion mutant of *L. pneumophila*.

## Results and Discussion

### Global transcriptional response of hMDMs to infection by wild type and an *ankB* mutant of *L. pneumophila*

We utilized the *ankB* mutant, that resides within an ER-derived LCV, similar to the wild type strain [15], as a genetic tool to determine if the transcriptional response of hMDMs to infection by *L. pneumophila* was dependent on intra-vacuolar proliferation. In order to examine the global transcriptional response of hMDMs to infection by either wild type or *ankB* mutant *L. pneumophila*, RNA from uninfected or infected hMDMs was isolated at 8 h post-infection and examined by microarray analysis. Interestingly, even though the *ankB* mutant fails to proliferate in hMDMs, the overall global transcriptional response of hMDMs to the mutant strain was similar to infection by the wild type strain (S1 Table). This result is likely due to Dot/Icm translocation-dependent activation of the global transcription factor NF- κB by *L. pneumophila* infection of human macrophages, which drives major changes in macrophage gene transcription [15], [28], [29], [30], [38], [39], [40], [41], [42], [43]. In addition, the ‘effector triggered response’ in C57BL/6J bMDMs, which is mediated by 5 translocated effectors that inhibit protein synthesis [26], are shared by the wild type strain and the *ankB* mutant. Therefore, analyses were focused on the shared transcriptional response of hMDMs to wild type and *ankB* mutant infection. The top 40 genes that showed the greatest differential expression in response to wild type or the *ankB* mutant are shown in Table 1. The up-regulated genes primarily encode mediators of the immune response such as IL23A, IL10 and TNF (Table 1), many of which but not all, have been shown previously to be up-regulated in C57BL/6J bMDMs infection by *L. pneumophila* Lp02 [25], [26]. The most down-regulated genes encode proteins with diverse cellular functions (Table 1). APOBEC3A was the most down-regulated genes in hMDMs infected with *L. pneumophila*. This gene encodes a cytidine deaminase that plays a key role in restriction of retroviruses by converting cytidine bases in viral DNA to uridine [44]. It is not currently known if APOBEC3A can affect DNA of intracellular bacterial pathogens, but it appears *L. pneumophila* actively protects itself from this host enzyme through down-regulation of its transcription. GAPT, the second most down-regulated gene in hMDMs infected with *L. pneumophila* binds to Grb2 and plays a role in B-cell activation and maintenance [45]. To facilitate the analysis of the microarray data, MetaCore Pathway Enrichment analyses were performed.

**Table 1:** The top 40 up- and down-regulated genes in hMDMs infected by either wild type or an isogenic *ankB L. pneumophila* mutant strain compared to uninfected hMDMs at 8 h post-infection.

	Upregulated			Downregulated	
Gene	wild type	ankB	Gene	wild type	ankB
**RANBP3L**	24.9558	26.77	**APOBEC3A**	−2.1301	−2.73152
**EGR1**	20.546	24.0411	**GAPT**	−2.03252	−2.7872
**NR4A2**	17.8068	18.0274	**ACAA1**	−1.68363	−1.1802
**CCL20**	12.9572	13.7933	**CD177**	−1.61745	−1.56168
**RHCG**	12.7492	15.4015	**MIR186**	−1.61347	−1.5868
**PMAIP1**	11.8732	12.7965	**PSG3**	−1.57598	1.05404
**IL23A**	9.56236	12.0511	**MSL3L2**	−1.57256	−1.37738
**GADD45B**	8.87087	10.1934	**LRRC25**	−1.57178	−1.88843
**HES1**	8.83049	10.3995	**CLEC4A**	−1.5458	−1.75417
**LIF**	8.79145	10.6728	**EFTUD1**	−1.53079	−1.38747
**FOS**	8.7718	8.89375	**SNORD49A**	−1.52728	−1.37486
**CSF2**	8.40756	10.9112	**INSIG2**	−1.50978	−1.34251
**IL10**	7.94675	8.5155	**LOC100133315**	−1.50807	−1.19079
**CXCL2**	7.55389	8.69098	**C5orf20**	−1.47832	−1.60939
**IL12B**	7.53365	10.1927	**CMKLR1**	−1.47262	−1.74118
**PTX3**	7.49085	9.93165	**SNORD5**	−1.46528	−1.4449
**IL6**	7.41431	7.90096	**CD209**	−1.46377	−1.05034
**TNF**	6.88512	7.34775	**C5orf44**	−1.45662	−1.43777
**E2F7**	6.83706	9.88067	**LOC221442**	−1.45215	−1.4062
**IL20**	6.72872	9.20687	**ZNF814**	−1.45034	−1.44363
**CLCF1**	6.67757	7.83769	**CLCN4**	−1.44066	−1.44317
**LOC440896**	6.5948	7.38741	**RAB42**	−1.43825	−1.36904
**ZFP36**	6.59423	8.02248	**C8orf44**	−1.43825	−1.44839
**MIR155**	5.77859	5.98857	**SUMO1P3**	−1.43729	−1.04598
**GADD45A**	5.51276	7.40294	**RASA4**	−1.43024	−1.74162
**KLF4**	4.73673	5.55325	**MIR142**	−1.42423	−1.76193
**CCL4**	4.73258	5.05042	**ZNF573**	−1.42362	−1.2825
**HIVEP2**	4.64881	5.47726	**AKR1C3**	−1.42287	−1.38282
**TNFSF9**	4.61437	5.90509	**FCGR3A**	−1.41971	−1.44462
**FOSB**	4.53659	4.5787	**CLEC6A**	−1.41775	−1.48098
**NFKBIZ**	4.52984	6.05722	**ZNF846**	−1.41227	−1.13047
**DENND4A**	4.46864	5.57978	**ZNF594**	−1.40638	−1.70581
**NIPAL4**	4.39544	5.99992	**CDKN3**	−1.40499	−1.39675
**TFPI2**	4.3389	4.61199	**LYPLAL1**	−1.40468	−1.38222
**GEM**	4.29366	5.00829	**SNORD42B**	−1.40304	−1.61795
**IFNG**	4.21353	2.68894	**FPR3**	−1.40273	−1.73894
**ZBTB10**	4.17859	4.82323	**C5orf54**	−1.40168	−1.88889
**OVOS**	4.17333	4.11822	**TRIM16**	−1.3968	−1.37838
**CXCL3**	3.99019	4.59161	**LOC344887**	−1.3964	−1.44194
**GPR109B**	3.9855	5.21438	**SNORD96A**	−1.3957	−1.36989

Numbers indicate fold change.

### Intra-vacuolar *L. pneumophila* triggers transcription of multiple immunologic pathways in hMDMs

**Figure 1:**
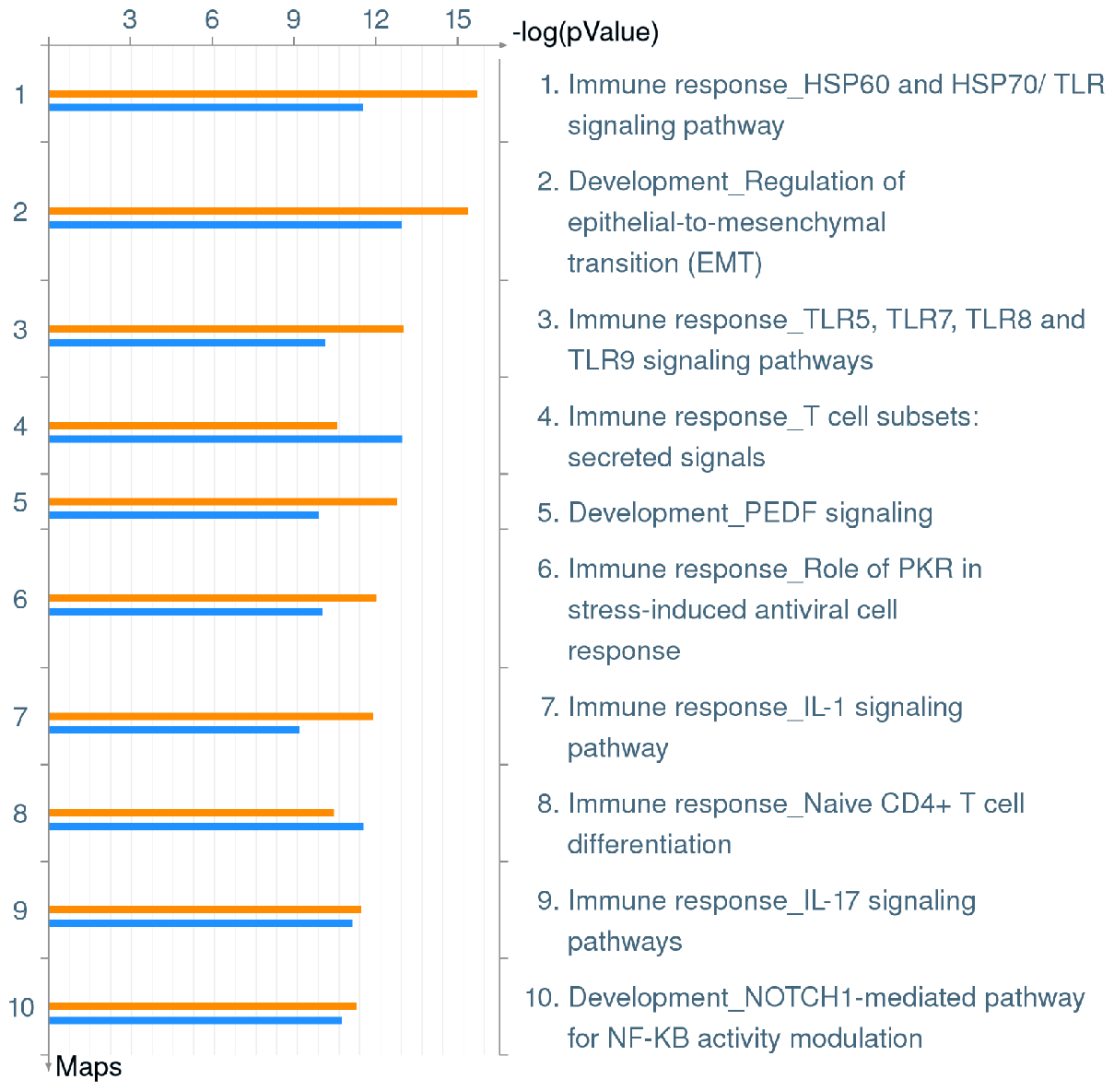
Top 10 Pathway Maps up-regulated in hMDMs upon infection by *L. pneumophila*. Metacore analysis of triplicate microarrays of wild type strain and *ankB* mutant-infected hMDMs at 8 h post-infection showing the top 10 Pathway Maps. The upper orange bar of each column represents wild type-infected hMDMs while the lower blue bar represents the *ankB* mutant-infected hMDMs.

MetaCore enrichment analysis of the microarray data revealed that several key immunological response pathways are the most significantly up-regulated during infection by the wild type strain or the *ankB* mutant of *L. pneumophila*. The top scoring Pathway Maps for both wild type and *ankB* mutant infected hMDMs are shown in Fig. 1 and individual genes affected by *L. pneumophila* infection in each of these Pathway Maps can be seen in S2 Table. The top three Pathway Maps include ‘Immune response HSP60 and HSP70/TLR’ (*p* value wild type 1.886^−16^, *ankB* 3.530^−12^), ‘Development_Regulation of epithelial-to-mesenchymal transition (EMT) signaling pathway’ (*p* value wild type 4.154^−16^, *ankB* 1.290^−13^) and ‘Immune response TLR5, TLR7, TLR8 and TLR9 signaling pathways’ (*p* value wild type 1.082^−13^, *ankB* 9.093^−11^) (Fig. 1, S2 Table). This indicates *L. pneumophila* instigates a robust innate immune response in infected hMDMs and this response is independent of intra-vacuolar proliferation.

**Figure 2:**
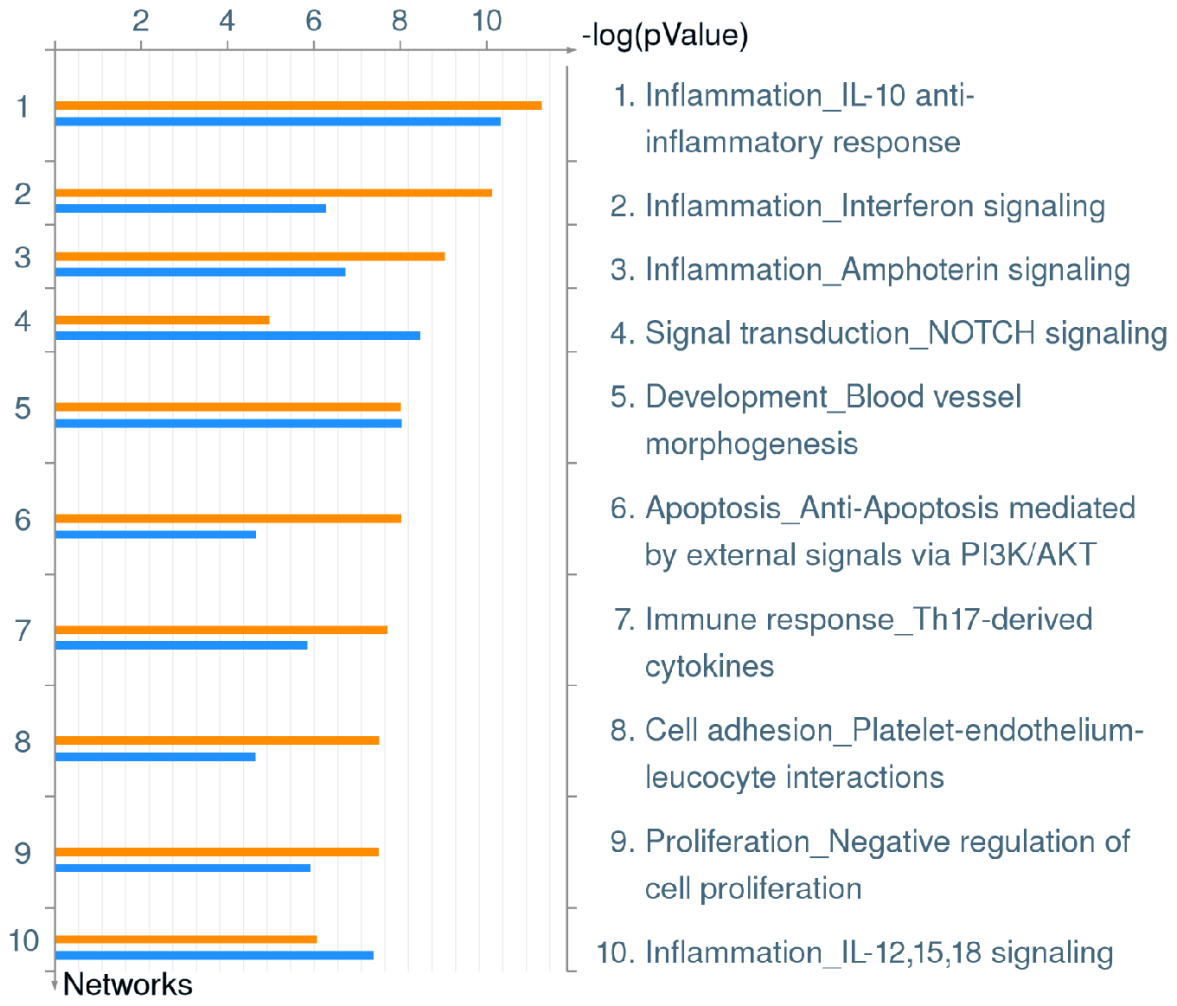
Top 10 Process Networks up-regulated in hMDMs upon infection by *L. pneumophila*. Metacore analysis of triplicate microarrays of wild type strain and *ankB* mutant-infected hMDMs at 8 h post-infection showing the top 10 Process Networks. The upper orange bar of each column represents wild type-infected hMDMs while the lower blue bar represents the *ankB* mutant-infected hMDMs.

A clearer overall impact of *L. pneumophila* infection on the hMDM global transcriptional response can be observed through enrichment of Process Networks, which combine the manually collated Pathway Maps and Gene Ontology (GO) processes to demonstrate major cellular processes. Genes in each of these process networks affected by *L. pneumophila* are listed in S2 Table. The data revealed that infection of hMDMs with either wild type or *ankB* mutant *L. pneumophila* induces a broad transcriptional response of genes involved in innate immunity. Five of the top ten Process Networks identified were associated with inflammation and the immune response (IL-10 anti-inflammatory response, interferon signaling, amphoterin signaling, IL-12,15,18 signaling and immune response to Th17-derived cytokines) (Fig. 2, S2 Table). *L. pneumophila* infection stimulates both pro-inflammatory Th1 and anti-inflammatory Th2 cytokine responses. Studies have shown that *L. pneumophila* stimulates the production of many pro-inflammatory cytokines including IL-1α, IL-1β, IL-6, IL-8, TNF-α, IFN-γ, IL-12, IL-17 and IL-18 by mouse bMDMs, human macrophage cell lines, murine models of *L. pneumophila* infection and in patients with Legionnaires' disease [28], [29], [30], [46], [47], [48], [49], [50], [51], [52], [53], [54], [55]. In addition, *L. pneumophila* stimulates the production of IL-10, the chief mediator of the Th2 response, which suppresses production of Th1 cytokines such as IFN-γ, promoting an anti-inflammatory response and allowing *L. pneumophila* to proliferate within hMDMs, primary human alveolar macrophages, U937 human macrophage cell line and mouse bMDMs [56], [57], [58], [59]. Therefore, it appears *L. pneumophila* endeavors to strike a balance between Th1 and Th2 responses and our microarray data clearly demonstrate that *L. pneumophila* modulates both pro- and anti-inflammatory pathways in hMDMs, and these host modulations are independent of intra-vacuolar proliferation.

Two Process Networks involved in cellular differentiation were also upregulated in hMDMs during infection by wild type or *ankB* mutant bacteria. The NOTCH signaling Process Network, which plays a key role in cell-cell communication during differentiation (*p* value wild type 1.238^−04^, *ankB* 3.857^−09^) and the Process Network defining blood vessel morphogenesis (*p* value wild type 3.503^−07^, *ankB* 1.044^−08^) were both up-regulated during wild type or *ankB* mutant infection (Fig. 2, S2 Table). Currently, little is known regarding these processes in relation to *L. pneumophila* infection.

*L. pneumophila* employs a multi-faceted approach to modulate host cell apoptosis, delaying death of the infected cell, enabling time for the bacteria to proliferate [39], [60], [61], [62], [63]. This is partially achieved through translocation of several effectors including SdhA, SidF, LegK1 and LnaB [64], [65], [66], [67] that either directly or indirectly modulates the apoptosis pathway. *L. pneumophila* also induces NF-κB dependent transcription of anti-apoptotic genes [38], [39] and this is at least dependent on the LegK1 effector, which directly phosphorylates the NF-κB inhibitor IκBα, promoting its proteasomal degradation [66], and on five effectors that block host cell protein synthesis that ultimately promote NF-κB dependent transcriptional activity [26]. The PI3K/Akt signaling pathway plays a crucial role in maintenance of eukaryotic cell survival through control of major cellular processes including glucose metabolism, protein synthesis, anti-apoptosis and phagocytosis [68]. Our microarray data show that the Process Network defining anti-apoptosis mediated by external signals via PI3K/Akt (*p* value wild type 1.075^−08^, *ankB* 2.859^−05^) is up-regulated in hMDMs infected with wild type or *ankB* mutant *L. pneumophila* (Fig. 2, S2 Table). Activation of the PI3K/Akt pathway is important in the phagocytosis of *L. pneumophila* by macrophages [69]. Therefore, our data implicate that upon phagocytosis, *L. pneumophila* reprograms diverse pathways in hMDMs to block apoptosis through activation of the PI3K/Akt pathway, in addition to the activity of various translocated effectors that promote NF-κB activity which prolongs cell survival [26], [39], [66], protecting the intracellular niche for *L. pneumophila*.

Infection of hMDMs with wild type or *ankB* mutant *L. pneumophila* also impacted genes involved in platelet-endothelium-leucocyte interactions (*p* value wild type 3.547^−08^, *ankB* 2.894^−05^) and regulation of cell proliferation (*p* value wild type 3.650^−08^, *ankB* 1.474^−06^) (Fig. 2, S2 Table). Infection of mouse bMDMs by the *L. pneumophila* Lp02 strain also induced genes associated with cell proliferation [25]. However currently, there are little data available to indicate the importance of these pathways in relation to *L. pneumophila* infection. Taken together, *L. pneumophila* infection of hMDMs up-regulates transcription of multiple immune responses, anti-apoptosis, cellular differentiation, proliferation and adhesion processes. This robust global transcriptional response in hMDMs is similar to that observed in infected bMDMs and U937 cells despite differences in genetic susceptibility and mammalian species [25], [26], [38]. Importantly, these global *L. pneumophila*-triggered host modulations are independent of intra-vacuolar proliferation of the bacteria.

### Repression of transcription of protein synthesis pathways in hMDMs independent of intra-vacuolar proliferation of *L. pneumophila*

**Figure 3:**
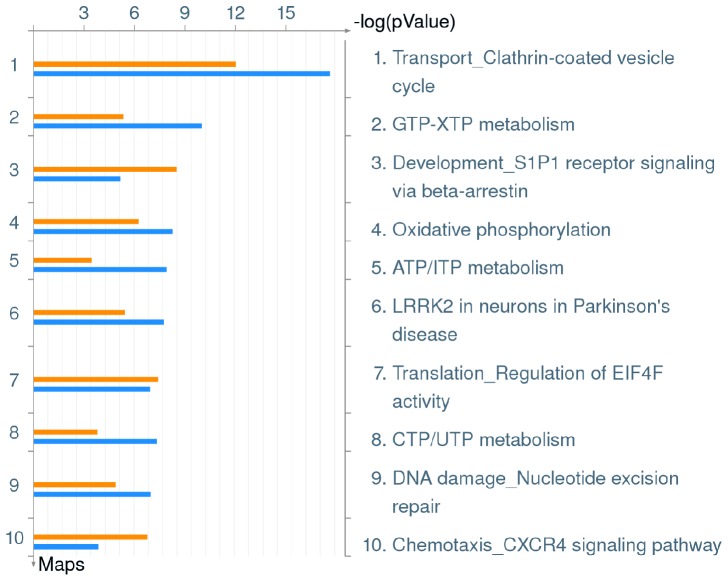
Top 10 Pathway Maps down-regulated in hMDMs upon infection by *L. pneumophila*. Metacore analysis of triplicate microarrays of wild type strain and *ankB* mutant-infected hMDMs at 8 h post-infection showing the top 10 Pathway Maps. The upper orange bar of each column represents wild type-infected hMDMs while the lower blue bar represents the *ankB* mutant-infected hMDMs.

In addition to analyses of cellular pathways up-regulated in hMDMs upon infection by *L. pneumophila*, down-regulated pathways were analyzed. Initial enrichment analysis for Pathway Maps revealed multiple host pathways that are down-regulated upon infection by both the wild type strain and the *ankB* mutant. These included clathrin-coated vesicle cycle, multiple nucleotide metabolism pathways, oxidative phosphorylation, EIF4F protein translation, S1P1 receptor signaling, LRRK2 signaling, CXCR4 signaling and nucleotide excision repair (Fig. 3, S3 Table). It is clear *L. pneumophila* infection of hMDMs down-regulates many pathways that have broad effects on the host cell. For example LRRK2 interacts with a wide range of signaling proteins including the MAPK family, and can modulate GTPase-activating and GTPase-exchange factors that affect vesicular trafficking and autophagy and altering this pathway may provide benefit for the pathogen [70]. Genes affected by *L. pneumophila* in these pathways are listed in S3 Table.

**Figure 4:**
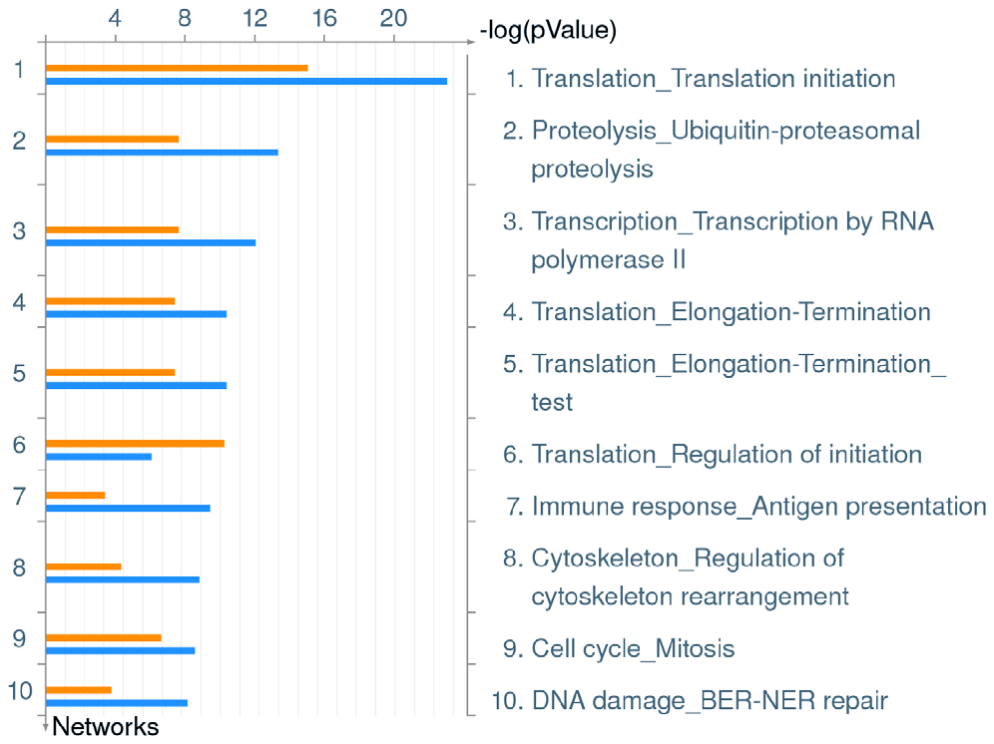
Top 10 Process Networks down-regulated in hMDMs upon infection by *L. pneumophila*. Metacore analysis of triplicate microarrays of wild type strain and *ankB* mutant-infected hMDMs 8 h post-infection showing the top 10 Process Networks. The upper orange bar of each column represents wild type-infected hMDMs while the lower blue bar represents the *ankB* mutant-infected hMDMs.

To develop a broad understanding of the effect of *L. pneumophila* infection in hMDMs in terms of down-regulated pathways, enrichment of down-regulated Process Networks was performed. Strikingly, *L. pneumophila* infection causes a broad repression of genes involved in protein translation in hMDMs, with 3 out of top 10 scoring Process Networks involved in protein translation initiation and termination (Fig. 4, S3 Table). *L. pneumophila* infection directly affects networks defining ‘Protein Translation Initiation’ (*p* value wild type 1.157^−15^, *ankB* 9.261^−24^) ‘Regulation of Initiation’ (*p* value wild type 9.097^−11^, *ankB* 1.527^−06^ and also ‘Elongation – Termination’ (*p* value wild type 6.960^−08^, *ankB* 6.962^−11^), indicating a significant and diverse impact on overall host protein translation (Fig. 4, S3 Table). Interestingly, infection of hMDMs with either wild type or *ankB* mutant *L. pneumophila* also negatively impacted the network defining ‘Transcription by RNA polymerase II’ (*p* value wild type 4.222^−08^, *ankB* 1.369^−12^) (Fig. 4, S3 Table). Genes directly impacted by *L. pneumophila* in these Process Networks can be seen in S3 Table. *L. pneumophila* is known to reduce protein translation in the host cell through the action of at least five translocated effectors [32], [33], [34], [35], [36]. It is possible that blocking host cell protein synthesis and transcription is beneficial for *L. pneumophila* through increasing cytosolic availability of amino acids in addition to promoting proteasomal degradation [19]. *L. pneumophila* relies heavily on amino acids as its primary source of carbon and energy and actively promotes increased host cytosolic amino acid concentrations through AnkB-dependent proteasomal degradation [19]. Similar to the wild type strain, the *ankB* mutant also reduced transcription of the protein synthesis pathways in hMDMs, but this mutant fails to replicate. This suggests any increase in bioavailability of amino acids due to reduced host protein translation plays only a minor role for *L. pneumophila* in terms of raising the levels of amino acids in hMDMs above the threshold needed for intra-vacuolar proliferation. *L. pneumophila* encodes five effectors that directly block host protein translation [32], [33], [34], [35], [36], and in non-permissive bMDMs derived from C57BL/6J mice leads to induction of the ‘effector-triggered response (ETR)’ characterized by increased transcription of IL23a and Gem [26]. In addition, *L. pneumophila*-dependent inhibition of host translation generates a frustrated MAP kinase response where many genes are transcribed but not translated, however a subset of pro-inflammatory cytokines including Il-1α and Il-1β can bypass this effect in a MyD88-dependent manner [37]. Transcription of both IL23a and Gem were amongst the top 40 highest expressed genes in hMDMs infected with *L. pneumophila* (Table 1), indicating the ETR phenotype also occurs in primary human macrophages, regardless of the genetic susceptibility or mammalian species of the host cell. Taken together, it appears *L. pneumophila* combines inhibition of protein translation pathway transcription and a direct effector-dependent inhibition of protein synthesis that leads to elevated expression of innate immunological pathways against pathogens in hMDMs.

### Global transcriptional down-regulation of host pathways in *L. pneumophila-*infected hMDMs

In addition to major impacts on gene transcription and protein translation, *L. pneumophila* infection negatively impacted several other major cellular processes. The second highest scoring down-regulated Process Network identified in *L pneumophila*-infected hMDMs defines ‘Ubiquitin-proteasomal proteolysis’ with *p* values of 4.243^−08^ and 6.605^−14^ for wild type and *ankB* mutant infection, respectively (Fig. 4, S3 Table). *L. pneumophila* hijacks the ubiquitin-proteasome system through multiple mechanisms to promote successful intracellular infection [19], [20], [21], [71], [72], [73]. The effector, LubX, mimics the function of eukaryotic U-box domain proteins to ubiquitinate the host cell kinase CDC2-like kinase 1 [71], though the consequences of LubX-mediated ubiquitination are currently unclear, it appears to be important for bacterial replication in A/J mouse macrophages [71]. LubX also functions as a ‘meta-effector’ by promoting the ubiquitination and subsequent proteasomal degradation of the SidH effector [72]. *L. pneumophila* harbors several effector proteins that encode the eukaryotic F-box domain [74], [75]. F-box domain proteins play an important role in directing target proteins to the SCF1 ubiquitin ligase complex to promote their ubiquitination [75]. In *L. pneumophila* strain Philadelphia-derived Lp02, the F-box effector LegU1 promotes the ubiquitination of the host chaperone protein BAT3, a protein that plays an important role in regulation of ER stress [21], [76], [77]. Another F-box effector protein, AnkB interacts with the host SCF1 ubiquitin ligase in the LCV membrane [22] and functions as a platform for the docking of lys^48^-linked polyubiquitinated proteins to the LCV membrane [18], [20] that are subsequently degraded by the host proteasome machinery, generating higher levels of cellular amino acids that are used by *L. pneumophila* to feed the TCA cycle [19]. *L. pneumophila* infection of bMDMs derived from A/J mice also reduces formation of dendritic cell aggresome-like structures (DALIS) that are enriched in ubiquitinated proteins and is dependent on the Dot/Icm T4SS [73]. DALIS formation is believed to protect ubiquitinated proteins from proteasomal degradation and serve as a pool of antigens available for downstream processing in dendritic cells [78]. Currently, the significance of the inhibition of DALIS formation in A/J mouse bMDMs by *L. pneumophila* is unclear.

In addition to effector-dependent modulation of host ubiquitination pathways, recognition of *L. pneumophila* ‘signatures’ by macrophages results in ubiquitination and subsequent proteasomal degradation of mTOR regulators [79]. This results in an enhanced pro-inflammatory cytokine response in macrophages [79]. It is clear that *L. pneumophila* actively hijacks the host ubiquitin/proteasome system at the protein level, however transcription of genes involved in the Process Network ‘ubiquitin-proteasomal proteolysis’ are also repressed by *L. pneumophila*. This suggests that hMDMs endeavor to limit the ability of *L. pneumophila* to affect the ubiquitin-proteasomal pathway at the transcriptional level albeit with limited success.

The Process Network that defines ‘Antigen presentation’ was also down-regulated in hMDMs by *L. pneumophila* infection (*p* value wild type 8.228^−04^, *ankB* 6.411^−10^), suggesting that even though a robust immune transcriptional response is induced, presentation of antigens is reduced (Fig. 4, S3 Table). The Process Network which encompasses ‘Regulation of cytoskeleton rearrangement’ was significantly down-regulated in hMDMs infected with *L. pneumophila* (*p* value wild type 9.401^−05^, *ankB* 2.536^−09^), and the related Process Network ‘Cell Cycle – Mitosis’ that shares many genes as ‘Regulation of cytoskeleton rearrangement’ was similarly affected in hMDMs infected with *L. pneumophila* (*p* value wild type 4.495^−07^, *ankB* 4.911^−09^) (Fig. 4, S3 Table). Two *L. pneumophila* translocated effectors have been shown to directly modulate the host cell cytoskeleton. The VipA effector functions as an actin nucleator and localizes to endosomes during *L. pneumophila* infection and likely interferes with organelle trafficking in the host cell [80]. In contrast to VipA, the Ceg14 effector inhibits actin polymerization through an uncharacterized mechanism [81]. Interestingly, protein translation in eukaryotic cells is tightly linked to the cytoskeleton [82]. We found that genes involved in protein synthesis and the cytoskeleton are down-regulated in infected hMDMs. Therefore, it will be interesting to determine if effectors that modulate the cytoskeleton have downstream effects on host protein synthesis. Conversely, it is possible effectors that inhibit protein synthesis [32], [33], [34], [36] may also impact the host cytoskeleton.

Our data show that infection of hMDMs with *L. pneumophila* reduce transcription of genes that define the Process Network ‘DNA damage – BER-NER repair’ (*p* value wild type 3.552^−04^, *ankB* 1.250^−08^), suggesting intracellular infection of *L. pneumophila* reduces the ability of hMDMs to repair DNA damage (Fig. 4, S3 Table). Currently, how this applies to *L. pneumophila* infection is unclear.

### Modulation of metabolic pathways in hMDMs by *L. pneumophila*

**Figure 5:**
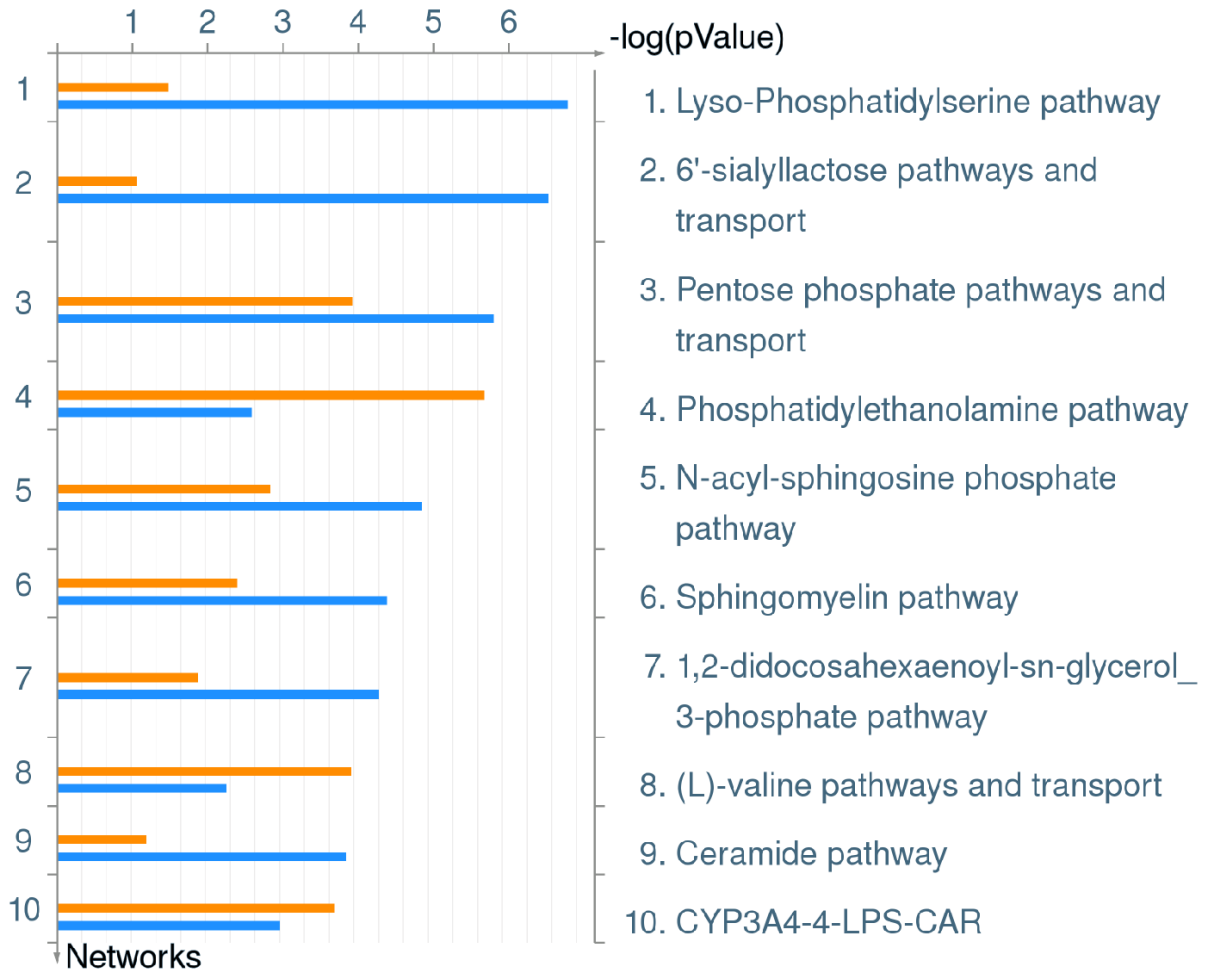
Top 10 Metabolic Pathways up-regulated in hMDMs upon infection by *L. pneumophila*. Metacore analysis of triplicate microarrays of wild type strain and *ankB* mutant-infected hMDMs 8 h post-infection showing the top 10 Metabolic Pathways. The upper orange bar of each column represents wild type-infected hMDMs while the lower blue bar represents the *ankB* mutant-infected hMDMs.

**Figure 6:**
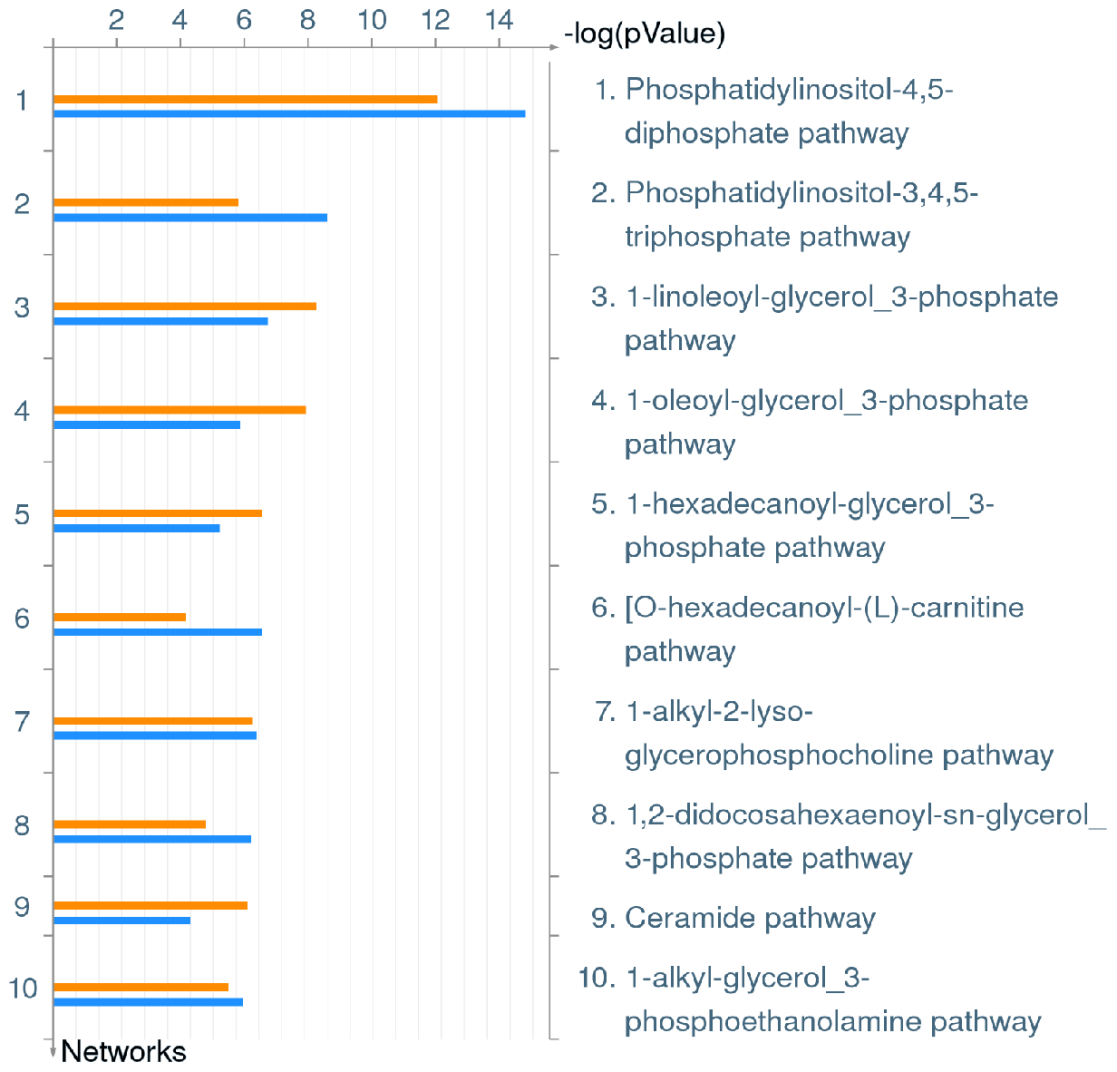
Top 10 Metabolic Pathways down-regulated in hMDMs upon infection by *L. pneumophila*. Metacore analysis of triplicate microarrays of wild type strain and *ankB* mutant-infected hMDMs 8 h post-infection showing the top 10 Metabolic Pathways. The upper orange bar of each column represents wild type-infected hMDMs while the lower blue bar represents the *ankB* mutant-infected hMDMs.

We have previously shown that infection of hMDMs by *L. pneumophila* triggers a rapid rise in intracellular amino acid concentrations above the threshold needed for *L. pneumophila* to utilize as a source of carbon and energy to power rapid bacterial growth [19]. Therefore, we examined which metabolic pathways in hMDMs are up- or down-regulated in response to infection by wild type or *ankB* mutant of *L. pneumophila*. The data show that infection of hMDMs by *L. pneumophila* triggers modulation of many metabolic pathways at the transcriptional level. The top 50 up- and down-regulated metabolic pathways are shown in S4 and S5 Tables and include broad alterations in lipid and amino acid metabolism and transport, as well as sugar metabolism (Figs. 5 and 6).

Interestingly, changes in diverse lipid metabolic pathways were the most pronounced metabolic alterations observed in hMDMs infected with by the wild type strain or the *ankB* mutant. Membrane lipids including sphingolipids and phosphoinositides regulate diverse cellular processes such as apoptosis, immunoregulation and migration, through interaction with specific cellular proteins that trigger diverse signaling cascades [83], [84]. Local concentrations of specific lipids act as thresholds for triggering cellular events. Therefore, lipid biosynthesis, degradation and transport are tightly controlled processes [84]. Our data show that *L. pneumophila* infection of hMDMs causes significant alterations in these diverse lipid metabolic pathways independent of bacterial replication, which in turn likely results in stimulation or repression of many host immunological pathways as demonstrated above. A number of *L. pneumophila* effectors have been shown to affect lipid pathways. For example, the effectors LecE and LpdA affect phospholipid metabolism in the host cell, while LegS2 affects sphingolipid metabolism [85], [86]. Hijacking phosphoinositides is a key virulence strategy of *L. pneumophila* [87]. These lipids play a central role in diverse processes including membrane trafficking, cytoskeleton and signaling pathways [84]. The LCV membrane is enriched for phosphatidylinositol-4-phosphate and several effectors anchor to this lipid to modulate biogenesis of the LCV [87]. Interestingly, the top down-regulated metabolic pathway in hMDMs infected with the wild type strain or the *ankB* mutant was the phosphatidylinositol-4,5-diphosphate pathway (Fig. 6, S5 Table). Phosphatidylinositol-4,5-diphosphate is hydrolyzed to phosphatidylinositol-4-phosphate via the enzyme OCRL1, which is localized to the LCV membrane [88]. This indicates that even though *L. pneumophila* hijacks phosphoinositide lipids during intracellular infection to modulate biogenesis of the LCV, host pathways for their generation are down-regulated in hMDMs during infection by *L. pneumophila*, and this is independent of intra-vacuolar proliferation.

The top up-regulated metabolic pathway in hMDMs infected by the wild type strain or the *ankB* mutant was the lyso-phosphatidylserine pathway (Fig. 5, S4 Table). Lyso-phosphatidylserine is a bio-active lipid that is increasingly shown to play a key role in initiation of acute inflammation and its subsequent resolution [89]. To date, the role of this bio-active lipid in macrophages during infection by intracellular bacteria remains unknown.

### Amino acid transporters

*L. pneumophila* has a strict requirement for amino acids that it satisfies by promoting elevated amino acid levels through proteasomal degradation of Lys^48^-linked polyubiquitinated proteins in the host cell cytosol [19]. Since *L. pneumophila* resides within a membrane bound compartment in its host cell, the bacteria must employ amino acid transporters to import cytosolic amino acids. The eukaryotic amino acid transporter SLC1A5, which transports a variety of neutral amino acids, is required for *L. pneumophila* replication in the Mono Mac 6 human macrophage cell line and has been identified via proteomics on LCVs isolated from RAW 264.7 mouse macrophages [90], [91]. The SLC7A5 and SLC3A2 amino acid transporters have also been identified by proteomic analysis to be present on LCVs isolated from RAW 264.7 mouse macrophages [91]. It is likely these transporters and others are recruited to the LCV to mediate the import of amino acids from the cytosolic milieu into the LCV lumen. Therefore, the microarray data were analyzed to determine if infection of hMDMs by *L. pneumophila* causes alterations in transcription of amino acid transporter genes. We observed that infection of hMDMs with *L. pneumophila* resulted in minor changes in transcription of amino acid transporters, with only 3 out of 45 genes showing statistically significant up- or down-regulation (Table 2). The cationic amino acid transporter SLC7A2 (CAT-2) was up-regulated 1.4 fold in *L. pneumophila* infected hMDMs and mediates the transport of arginine into macrophages [92], [93]. The mouse homolog of CAT-2, mCAT-2, is upregulated by *Salmonella* infection of mouse bMDMs and likely aids the import of arginine into the *Salmonella*-containing vacuole [94]. In addition, expression of SLC7A2 has been implicated in the ability of macrophages to mediate an innate immune response to *Helicobacter pylori* and *Leishmania* infection [93], [95], [96]. It is possible increased arginine transport in *L. pneumophila* infected hMDMs enhances the ability of the macrophage to clear infection by contributing to the production of nitric oxide by NOS2, or alternatively *L. pneumophila* hijacks this transporter to mediate arginine transport into the LCV. The glutamate transporter SLC1A2 (GLT1) was also induced 1.4 fold in hMDMs infected by *L. pneumophila* (Table 2). This transporter is primarily found in neural cells [97] but can be expressed in macrophages where it mediates uptake of glutamate [98], [99] and possibly hijacked by *L. pneumophila* to mediate transport of glutamate into the LCV during infection of hMDMs. SLC7A5 transports large neutral amino acids and has been implicated in the pathogenesis of *Salmonella* [100], and has been identified on the LCV by proteomics [91]. We observed a small but statistically significant 1.2 fold increase in SLC7A5 gene transcription in hMDMs infected with *L. pneumophila* (Table 2). It is likely *L. pneumophila* hijacks SLC7A5 to mediate import of amino acids into the LCV.

**Table 2:** Amino acid transporters up-regulated in hMDMs infected by *L. pneumophila*.

Gene Name	p-value	Fold-Change	Transporter function
SLC7A2	0.00079	1.43096	cationic L-amino acids
SLC1A2	0.000521	1.40055	glutamate, aspartate
SLC7A5	0.013758	1.20244	large neutral L-amino acids

Although up-regulation of the SLC amino acid transporters by *L. pneumophila* is modest, it is important to note that our infection conditions result in infection of ∼20% of the hMDMs in the monolayers. Therefore, it is expected that most transcriptional modulations throughout this study are much more pronounced in the infected hMDMs in the monolayers. However, small changes in transcription of certain genes in the infected cells may not be detectable in the whole cell population. Taken together, *L. pneumophila* up-regulates transcription of various host transporters during infection, which can contribute to the ability of this pathogen to import amino acids and other host molecules from the host cytosol into the LCV lumen.

In conclusion, we have shown that infection of hMDMs by *L. pneumophila* triggers robust transcription of inflammatory and immunological pathways whilst transcription of protein synthesis pathways is repressed. Furthermore, transcription of metabolic pathways in hMDMs is significantly altered by *L. pneumophila* infection and in particular lipid metabolism. Importantly, this global host cell response is independent of intra-vacuolar bacterial proliferation and validates findings of other studies that used bMDMs and human macrophage cells lines. Taken together, it is clear that human macrophages alter their transcriptional landscape in response to infection by *L. pneumophila* in an endeavor to mount a successful immune response to the pathogen regardless if the bacterium is able to proliferate intracellularly. Finally, the microarray data presented will be a useful resource for the research community in understanding global and complex *L. pneumophila*-hMDM interactions.

## Supporting Information

S1 Table The total processed microarray data of hMDMs uninfected or infected with wild type or *ankB* mutant *L. pneumophila* at 8 h post-infection.
(XLSX)

S2 Table Metacore enrichment analysis of hMDMs infected with wild type or *ankB* mutant *L. pneumophila* at 8 h post-infection showing up-regulated pathways.
(XLS)

S3 Table Metacore enrichment analysis of hMDMs infected with wild type or *ankB* mutant *L. pneumophila* at 8 h post-infection showing down-regulated pathways.
(XLS)

S4 Table Metacore enrichment analysis of hMDMs infected with wild type or *ankB* mutant *L. pneumophila* at 8 h post-infection showing up-regulated metabolic pathways.
(XLS)

S5 Table Metacore enrichment analysis of hMDMs infected with wild type or *ankB* mutant *L. pneumophila* at 8 h post-infection showing down-regulated metabolic pathways.
(XLS)
